# Discrimination of *Escherichia coli, Shigella flexneri*, and *Shigella sonnei* using lipid profiling by MALDI‐TOF mass spectrometry paired with machine learning

**DOI:** 10.1002/mbo3.1313

**Published:** 2022-08-25

**Authors:** Jade Pizzato, Wenhao Tang, Sandrine Bernabeu, Rémy A. Bonnin, Emmanuelle Bille, Eric Farfour, Thomas Guillard, Olivier Barraud, Vincent Cattoir, Chloe Plouzeau, Stéphane Corvec, Vahid Shahrezaei, Laurent Dortet, Gerald Larrouy‐Maumus

**Affiliations:** ^1^ Faculty of Natural Sciences, Department of Life Sciences, MRC Centre for Molecular Bacteriology & Infection Imperial College London England; ^2^ Faculty of Natural Sciences, Department of Mathematics Imperial College London England; ^3^ CHU de Bicêtre, Laboratoire de Bactériologie‐Hygiène Assistance Publique des Hôpitaux de Paris Le Kremlin‐Bicêtre France; ^4^ INSERM UMR 1184, Team RESIST, Faculté de Médecine Université Paris‐Saclay Le Kremlin‐Bicêtre France; ^5^ Centre National de Référence de la Résistance aux Antibiotiques Le Kremlin‐Bicêtre France; ^6^ Service de Microbiologie, Assistance Publique‐Hôpitaux de Paris, Hôpital Necker Enfants‐Malades AP‐HP Centre‐Université de Paris Paris France; ^7^ Service de Biologie Clinique Hôpital Foch Suresnes France; ^8^ Université de Reims‐Champagne‐Ardenne, Inserm UMR‐S 1250 P3Cell, SFR CAP‐Santé, Laboratoire de Bactériologie‐Virologie‐Hygiène, Hospitalière‐Parasitologie‐Mycologie, Hôpital Robert Debré CHU Reims Reims France; ^9^ CHU Limoges, Service de Bactériologie‐Virologie‐Hygiène, CIC1435, INSERM 1092 Université de Limoges, UMR Limoges France; ^10^ Service de Bactériologie‐Hygiène CHU de Rennes Rennes France; ^11^ Service de Bactériologie et d'Hygiène hospitalière, Unité de microbiologie moléculaire et séquençage CHU de Poitiers Poitiers France; ^12^ Université de Nantes, CHU Nantes, Service de Bactériologie et des Contrôles Microbiologiques, INSERM, INCIT UMR 1302 F‐ 44000 Nantes France

**Keywords:** identification, lipids, MALDI, *Shigella*

## Abstract

Matrix‐assisted laser desorption/ionization‐time of flight mass spectrometry (MALDI‐TOF MS) has become a staple in clinical microbiology laboratories. Protein‐profiling of bacteria using this technique has accelerated the identification of pathogens in diagnostic workflows. Recently, lipid profiling has emerged as a way to complement bacterial identification where protein‐based methods fail to provide accurate results. This study aimed to address the challenge of rapid discrimination between *Escherichia coli* and *Shigella* spp. using MALDI‐TOF MS in the negative ion mode for lipid profiling coupled with machine learning. Both *E. coli* and *Shigella* species are closely related; they share high sequence homology, reported for 16S rRNA gene sequence similarities between *E. coli* and *Shigella* spp. exceeding 99%, and a similar protein expression pattern but are epidemiologically distinct. A bacterial collection of 45 *E. coli*, 48 *Shigella flexneri*, and 62 *Shigella sonnei* clinical isolates were submitted to lipid profiling in negative ion mode using the MALDI Biotyper Sirius® system after treatment with mild‐acid hydrolysis (acetic acid 1% v/v for 15 min at 98°C). Spectra were then analyzed using our in‐house machine learning algorithm and top‐ranked features used for the discrimination of the bacterial species. Here, as a proof‐of‐concept, we showed that lipid profiling might have the potential to differentiate *E. coli* from *Shigella* species using the analysis of the top five ranked features obtained by MALDI‐TOF MS in the negative ion mode of the MALDI Biotyper Sirius® system. Based on this new approach, MALDI‐TOF MS analysis of lipids might help pave the way toward these goals.

## Graphical abstract

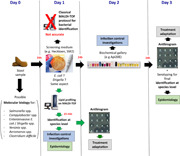

## 1 INTRODUCTION

There are four commonly recognized *Shigella* species (*Shigella boydii, Shigella dysenteriae, Shigella flexneri*, and *Shigella sonnei*), all of which may cause the well‐characterized disease known as shigellosis (Niyogi, 2005). In contrast, *Escherichia coli* strains in the human gut are typically commensal, although some pathovars can cause diarrhea. Shigellosis is endemic throughout the world and is responsible for nearly 165 million cases of severe dysentery each year (Kotloff et al., 1999; Niyogi, 2005). Since shigellosis is highly communicable (<100 viable cells can produce disease in healthy adults), it is a serious health concern at childcare centers and in developing countries with poor sanitation conditions. For example, in the United States, approximately 14,000 cases of shigellosis occur each year, with *S. flexneri* and *S. sonnei* identified as the predominant pathogens (Khalil et al., 2018). The Shiga‐toxin‐producing species *S. dysenteriae*, although infrequently isolated in the United States, may produce a more‐serious disease that can be fatal if left untreated.

**Figure A1:**
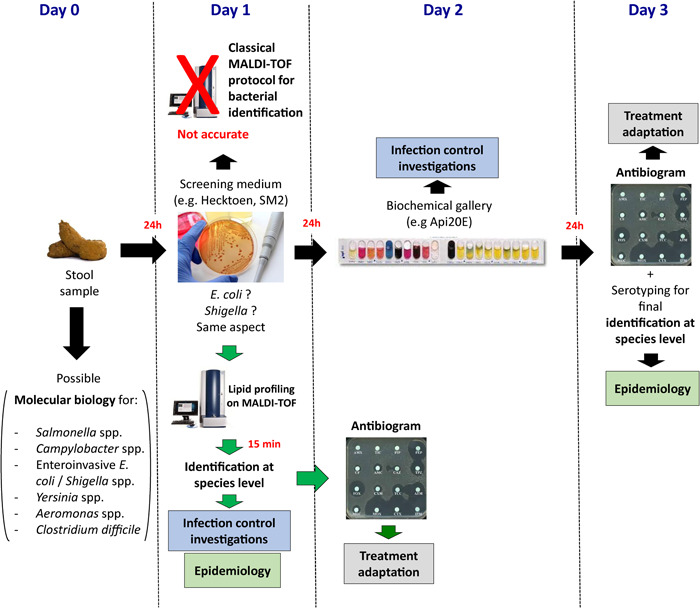
Workflows for the identification of shigellosis in a clinical microbiology laboratory. The routine workflow is represented by a black arrow while the lipid profiling identification workflow is represented by green arrows. Combined with a machine learning algorithm, lipid profiling by routine MALDI in the negative ion mode might have the potential to differentiate *Escherichia coli* from *Shigella* species.

*Shigella* species and *E. coli* are very closely related Gram‐negative bacteria belonging to the *Enterobacterales*. Phenotypically, *Shigella* species and *E. coli* share many common characteristics; genotypically, they could be considered the same species but present different infectiousness and clinical outcomes (Halimeh et al., 2021; Kaper et al., 2004; Pupo et al., 2000; van den Beld et al., 2019). Due to this close relatedness, the differentiation of *Shigella* species from *E. coli* can be difficult and time‐consuming. Nowadays, the diagnosis of shigellosis is based on the isolation of the pathogen from stool culture on conventional screening media, biochemical assays, and molecular detection such as 16S rRNA sequencing and/or amplification of the invasion plasmid antigen H (ipaH) (de Boer et al., 2010; Schaumburg et al., 2021; Van Lint et al., 2016; Vu et al., 2004; Zimmermann et al., 2020). The antibiotic treatment has to be implemented only if a true pathogen (i.e., *Shigella*) is identified, not if only commensal *E. coli* are isolated. Unfortunately, both *Shigella* and *E. coli* can grow on screening media. Currently, methods based on biochemical tests and serotyping are preferred for the discrimination of these species. However, these approaches may have suboptimal diagnostic performance as they are slow, relying on multiple‐step methods of culturing on selective agar, slide agglutination tests, and the use of commercial biochemical identification kits. In addition, *Shigella* species and *E. coli* are undistinguishable using molecular methods such as sequencing the 16S rRNA gene or molecular syndromic panel (usually a detection of the invasin gene *inv* i.e. common to *Shigella* spp. and enteroinvasive *E. coli* isolates) (Schaumburg et al., 2021; Zimmermann et al., 2020) as well as routine matrix‐assisted laser desorption/ionization‐time of flight mass spectrometry (MALDI‐TOF MS) (van den Beld et al., 2022). Indeed, protein‐based MALDI‐TOF MS, which is now the gold standard for bacterial identification in clinical microbiology laboratories, is unable to provide accurate differentiation of *Shigella* spp. and *E. coli*, likely due to their similar protein profiles (Devanga Ragupathi et al., 2018). Despite few studies reporting the possibility to discriminate *Shigella* spp. from *E. coli* using classical MALDI‐TOF MS with a specific reference library (Paauw et al., 2015) or algorithm for peaks interpretation (Khot & Fisher, 2013), these methods have never been implemented in routine testing (van den Beld et al., 2022). Accordingly, definitive discrimination between *E. coli* and *Shigella* spp. still relies on biochemical characters assessed in an additional 24 h using a biochemical gallery (e.g., API20E strip, Vitek®2 GN identification card). Then, serotyping can be performed to definitively discriminate between the four species: *S. boydii, S. dysenteriae, S. flexneri*, and *S. sonnei* (Figure A1). Despite literature showing that some lipids such as lipid A and LPS composition in *Shigella* species display some differences like numbers of acylation or presence of phosphoethanolamine groups (Casabuono et al., 2012), a lipid‐based MALDI‐TOF MS method had not yet been attempted as a rapid diagnostic tool.

This study aims to accelerate the application of routine MALDI‐TOF MS to address the public health challenge of the rapid discrimination between *Shigella* spp. and *E. coli*. To do so, we have explored the use of lipid profiling to discriminate between the closely related species of *E. coli* and the most prevalent Shigella species (*S. sonnei* and *S. flexneri*), for which speed of diagnostics is crucial to treat the patient and prevent and control outbreaks. Analysis using MALDI‐TOF MS in the negative‐ion mode combined with a machine learning algorithm demonstrated that it is possible to tell apart *E. coli, S. flexneri*, and *S. sonnei* using lipid profiles.

## 2 MATERIALS AND METHODS

### 2.1 Bacterial strains

A bacterial collection of 45 *E. coli* strains, 48 *S. flexneri* strains, and 62 *S. sonnei* strains was analyzed. These clinical isolates were recovered from stool samples of diarrheic patients who were admitted or hospitalized in eight different French hospitals. Before the analysis conducted in this study, isolates had been classified into species by standard biochemical and serotyping methods. These methods involved multiplex PCR (Seegene) on patient stool samples to search for the most common pathogens responsible for febrile diarrhea such as *Salmonella* spp., *Campylobacter* spp., enteroinvasive *E. coli/Shigella* spp., *Aeromonas* spp., and *Clostridium difficile*. For positive results, stool samples were isolated on adequate screening medium. Accordingly, if a positive signal was obtained by multiplex PCR for enteroinvasive *E. coli/Shigella* spp., a *Salmonella/Shigella* agar (bioMérieux, la Balme les Grottes, France) and a Hecktoen agar (bioMérieux) were used for culture. On colonies that cultured after 24 h incubation, identification was performed using an API20E biochemical strip (bioMérieux) or Vitek®2 GN identification card (bioMérieux), allowing discrimination between *E. coli* and *Shigella* spp. Then, serotyping was performed to definitively discriminate between the four species: *S. boydii, S. dysenteriae, S. flexneri*, and *S. sonnei*. In the routine workflow, if the API20E biochemical strip or the Vitek2 identified a sample as *E. coli*, multiplex PCR (syndromic molecular panel) might be performed on the bacterial colony to verify if this *E. coli* isolate corresponds to an enteroinvasive strain (acquisition of the *Shigella* invasin gene *inv*).

### 2.2 Sample preparation for lipid profiling

One bacterial colony was resuspended in 100 µL of water. This bacterial suspension was centrifuged at ×1000*g* for 10 min, then washed twice with 200 µL ddH_2_O. The pellet was resuspended in 100 µL of acetic acid 1% v/v and incubated in a PCR machine (T100 Thermal Cycler, Bio‐Rad) at 98°C for 15 min. After incubation, the pellet was washed twice with 200 µL ddH_2_O and then resuspended in 20 µL ddH_2_O. A volume of 0.4 µL of the hydrolyzed sample was mixed with a 1.2 µL Norharmane matrix (10 mg/mL, 9:1 chloroform/methanol, v/v) on a MALDI target plate named MSP 96 target polished steel BC (Bruker Part‐No. 8280800). The bacterial suspension and matrix were mixed directly on the target by pipetting and then dried gently under a stream of air.

### 2.3 MALDI‐TOF MS analysis

The spectra were recorded in the linear negative‐ion mode (laser intensity 95%, ion source 1 = 10.00 kV, ion source 2 = 8.98 kV, lens = 3.00 kV, detector voltage = 2652 V, pulsed ion extraction = 150 ns) using MALDI Biotyper Sirius® system (Bruker Daltonics). Each spectrum corresponded to an ion accumulation of 5000 laser shots randomly distributed on the spot for the range *m*/*z* 1000 to *m*/*z* 2500. The spectra obtained were processed with default parameters using FlexAnalysis v.3.4 software (Bruker Daltonics).

### 2.4 Pre‐processing of lipid spectra data

The bioinformatics analysis pipeline used R version 4.1.2. The method described here used code adapted from a study by Gibb & Strimmer (Gibb & Strimmer, 2015). “MALDIquant” (version 1.21) and “MALDIquantForeign” (version 0.13) packages were used to pre‐process the spectra data for all *E. coli, S. sonnei*, and *S. flexneri* samples. First, a square root transformation (sqrt) was performed on the intensities of the spectra. The intensity values were then smoothed using the Savitzky–Golay method (Steinier et al., 1972). The baseline of the mass spectrometry data was estimated and then removed using the statistics‐sensitive nonlinear iterative peak‐clipping (SNIP) algorithm. Intensity values were normalized using the total ion current method then spectra were aligned. A signal‐to‐noise ratio of 3 (SNR = 3) and a half window size of 20 (HWS = 20) were used to detect peaks above the defined threshold in the mass spectrometry data. Following this, the peak binning function was used to look for similar peaks across different spectra and equalize their mass. Finally, peaks that occurred infrequently within the same species group were removed from the data. After this pre‐processing, the result was a two‐dimensional feature matrix containing peak intensity information for the spectra of all samples.

### 2.5 Machine learning

After the above pre‐processing workflow, the feature matrix was converted into both a naïve binary absence‐presence matrix (replaced non‐negative and missing value with 1 and 0 respectively in feature matrix, true labels were not utilized) and a dichotomized binary matrix (For each feature (*m*/*z*), a threshold is determined by considering true labels. Intensities above that threshold will be set to 1, otherwise 0, via R packages “binda” [version 1.0.4] [Gibb & Strimmer, 2015]). Hierarchical clustering was applied to the naïve binary feature matrix to figure out if different species can be separated in an unsupervised manner.

Binary discriminant analysis was then applied to the dichotomized binary feature matrix to identify and rank the most differentially expressed peaks across the spectra and ascertain whether any of these peaks from the lipid profiles could be used for cla184‐ss prediction, i.e., to determine whether the spectra belonged to a sample of *E. coli, S. sonnei*, or *S. flexneri* (by computing *t*‐scores between the group means (in each species) and the pooled mean (across species). The top‐ranked peaks were used to test their class prediction ability. Data were further split into training and testing data (randomly picked 70% of all samples for training and the rest for testing) to study the robustness of the top‐ranked features in terms of classification of the three species.

## 3 RESULTS

### 3.1 *E. coli, S. sonnei*, and *S. flexneri* showed distinct lipid profiles in the range *m/z* 1700–1950

**Figure 1:**
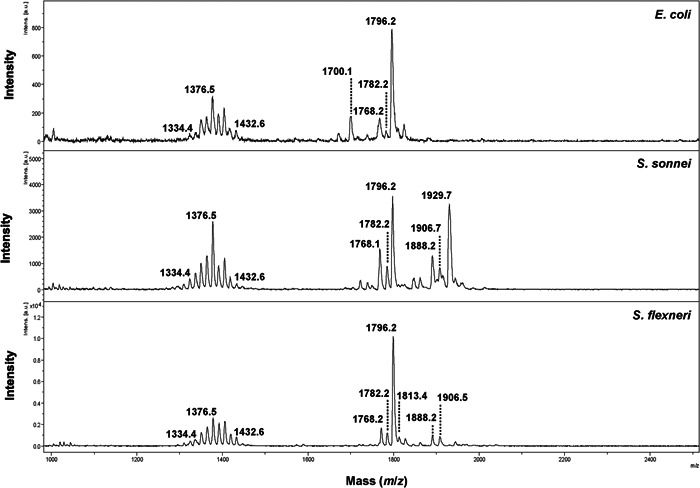
Linear negative ion mode mass spectra of *Escherichia coli* (top panel), *Shigella sonnei* (middle panel), and *Shigella flexneri* (bottom panel).

To assess the use of lipid profiling to discriminate *E. coli, S. sonnei*, and *S. flexneri* in a routine MALDI Biotyper Sirius® system, we tested a panel of 45 *E. coli*, 48 *S. flexneri*, and 62 *S. sonnei* clinical isolates. The samples were prepared to enrich membrane lipids as described earlier and the mass spectra were recorded in the linear negative ion mode. The range *m*/*z* 1000 to *m*/*z* 2500 was chosen as it gave spectra with the highest signal‐to‐noise (S/N) (>3) and mass resolution (>200), suitable for following data analyses. The range of interest (*m*/*z* 1000 to *m*/*z* 2500) in the *E. coli* spectrum (Figure 1, top panel) was dominated by two sets of peaks between *m*/*z* 1334.4 and *m*/*z* 1432.6 and between *m*/*z* 1700.1 and *m*/*z* 1800 assigned to cardiolipins and bisphosphorylated hexa‐acyl lipid A, respectively (Casabuono et al., 2012; Krokowski et al., 2018; Lindberg et al., 1991; Paciello et al., 2013). The major peak at *m*/*z* 1796.2 corresponds to hexa‐acyl diphosphoryl lipid A containing four 3‐OH‐C14:0 acyl groups, one C14:0 acyl group, and one C12:0 acyl group referred to as native lipid A (Casabuono et al., 2012; Lindberg et al., 1991; Paciello et al., 2013). In *S. sonnei* and *S. flexneri* (Figure 1), there were also two sets of peaks between *m*/*z* 1334.4 and *m*/*z* 1432.6 and between *m*/*z* 1700.1 and *m*/*z* 1950 assigned to cardiolipins and bisphosphorylated hexa‐acyl lipid A, respectively. However, despite the presence of similar peaks, differences were observed between the mass spectra generated from *E. coli, S. sonnei*, and *S. flexneri* in the mass range between *m*/*z* 1700 and *m*/*z* 1950. Based on this observation, we decided to combine the lipid profiles with machine learning to discriminate between the spectra of *E. coli, S. sonnei*, and *S. flexneri* samples.

### 3.2 Machine learning allows discrimination of *E. coli, S. sonnei*, and *S. flexneri* clinical isolates

**Figure 2:**
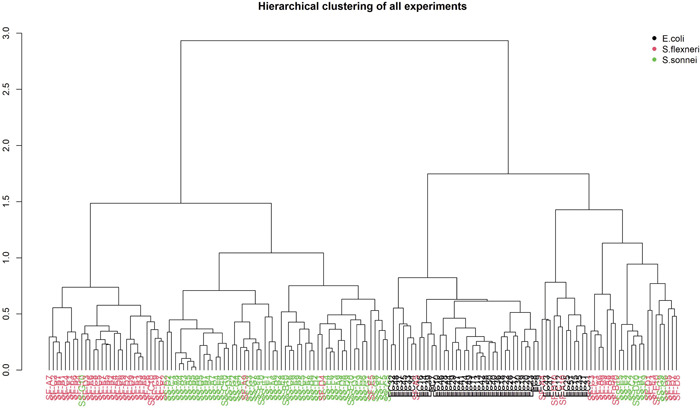
Dendrograms showing hierarchical clustering of *Escherichia coli, Shigella sonnei*, and *Shigella flexneri* samples. Black indicates *E. coli* isolates, red indicates *S. flexneri* isolates, and green indicates *S. sonnei* isolates. Clustering of species using the naïve binary absence‐presence matrix.

Following the first workflow for pre‐processing the data (Tang et al., 2019) (see details in pre‐processing of lipid spectra data section above), the lipid profiles already led to the clustering of three different entire lipid profiles in an unsupervised manner (Figure 2). Then, we identified top‐ranked peaks and validated their robustness in this classification.

**Figure 3:**
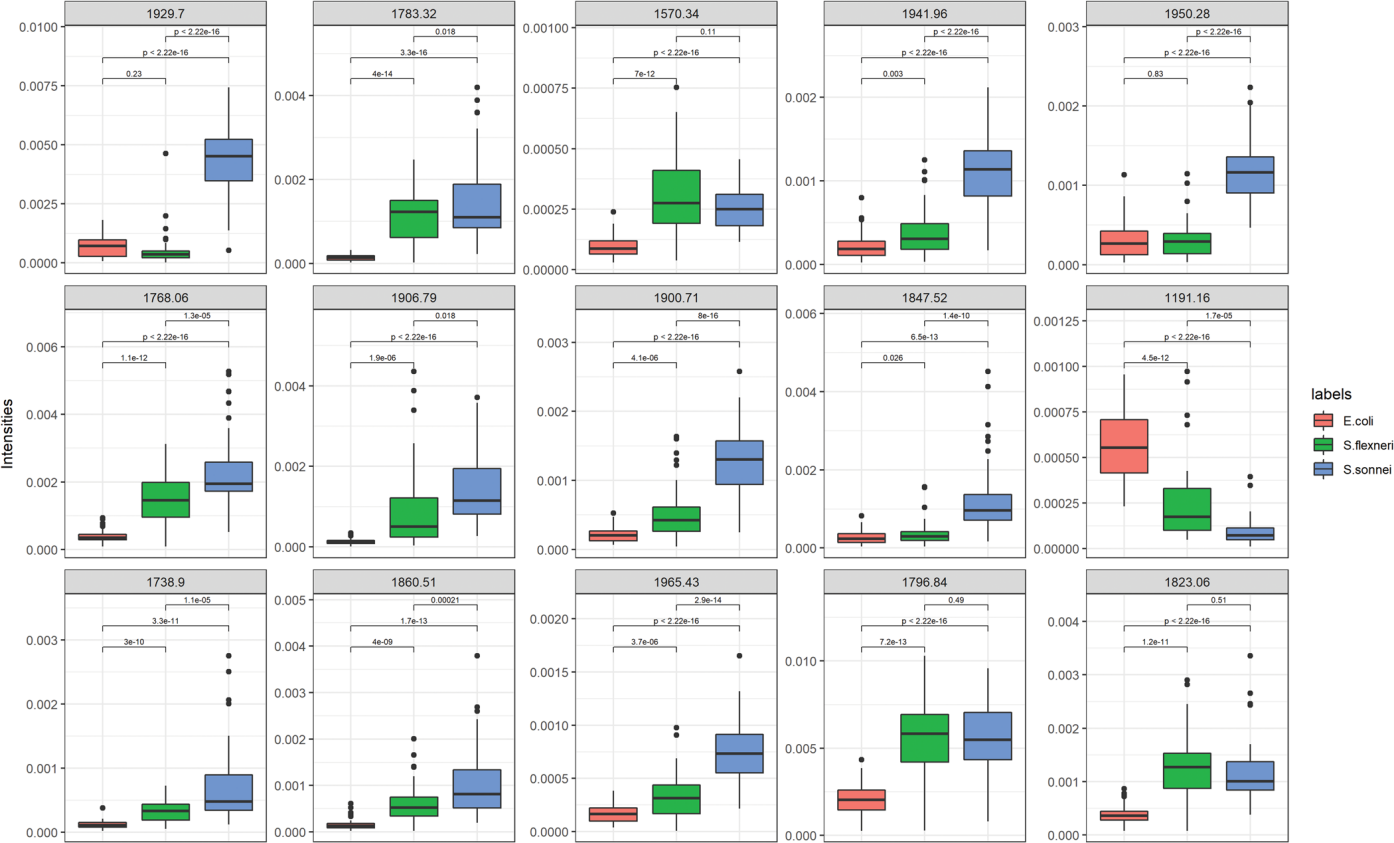
Top 15 ranked features reported from “binda” (ranked from left to right, from top to bottom). *p*‐values come from the *t*‐test.

The dichotomized matrix was used for extracting top‐ranked peaks (Table A1) based on multi‐class discriminant analysis using binary predictors in a supervised manner (Gibb & Strimmer, 2015). The intensities of the 15 top‐ranked peaks reported from “binda” efficiently distinguished *E. coli, S. sonnei*, and *S. flexneri* (Figure 3).

### 3.3 Validation of the robustness of top‐ranked peaks via supervised learning and using randomly selected features as controls

**Figure 4:**
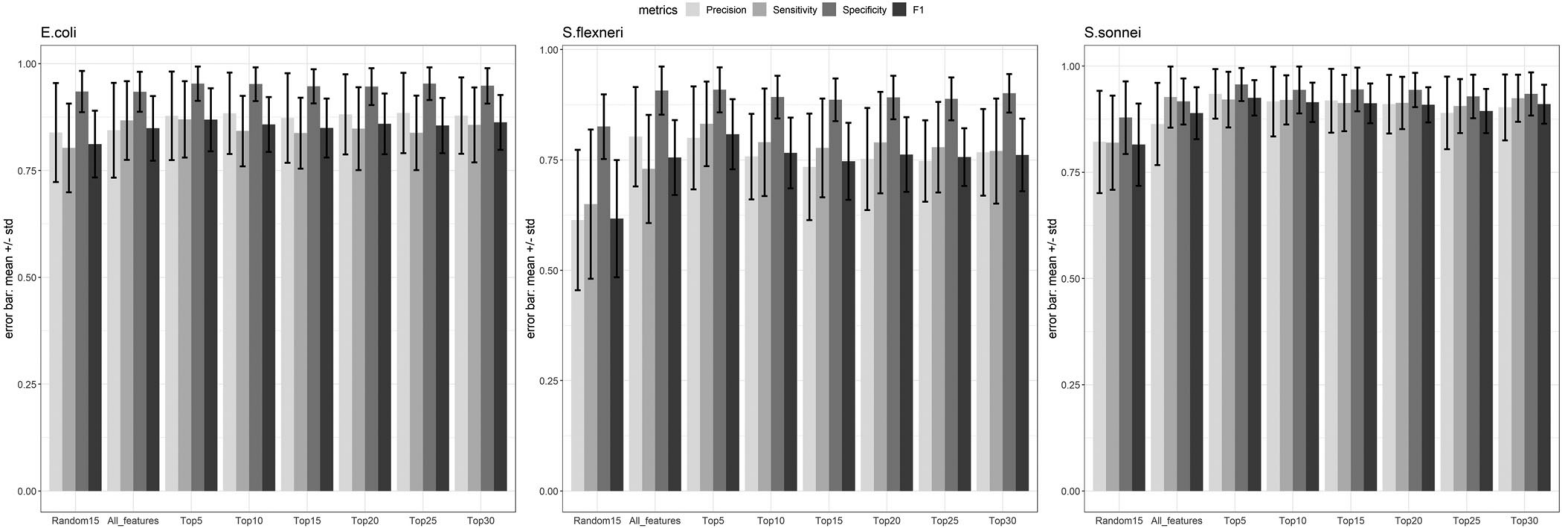
Bar plots showing accuracy values for species prediction (between *Escherichia coli* and combination of *Shigella flexneri* and *Shigella sonnei*) using top‐ranked features; all features and randomly selected features. The accuracy values come from the analysis being repeated 100 times of splitting training and testing data and random selection of peaks for control.

**Figure 5:**
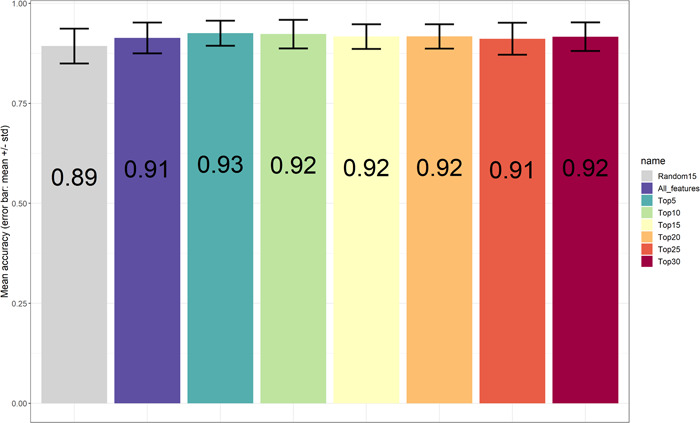
Barplots showing accuracy values for species prediction (between *Shigella flexneri* and *Shigella sonnei*) using top‐ranked features; all features and randomly selected features. The accuracy values come from the analysis being repeated 100 times by splitting training and testing data and random selection of peaks for control.

**Figure 6:**
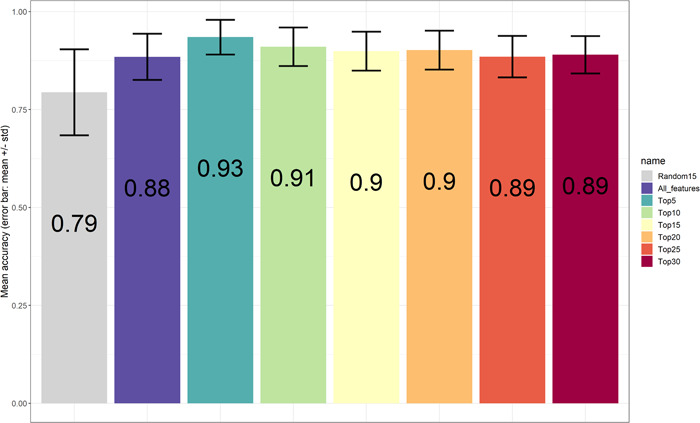
Classification metrics including precision, sensitivity, specificity, and F1

To validate the robustness of the top‐ranked peaks in terms of classification of the three bacterial species, data were further separated into training and testing data (70% out of 155 samples for training). The random separation of training and testing data was repeated 100 times. Various numbers of top‐ranked peaks including all peaks (89 in total) and one set of randomly selected 15 peaks were used as controls. Consistent with a previous report from Tang et al. (2019) only minor differences could be found using either top‐ranked peaks or whole peaks with respect to accuracy rates. Just a subset of top‐ranked peaks would be enough for this classification problem (Conrad et al., 2017; Gibb & Strimmer, 2015). Using top‐ranked peaks, we were able to achieve relatively higher performance in terms of four metrics precision, sensitivity, specificity, and F1 score for identifying *E. coli* and *Shigella* spp. In addition, when simply looking at accuracy rates based on the comparison between *E. coli* and *Shigella* spp. (*S. flexneri* + *S. sonnei*, around 0.9, Figure 4) or between *S. flexneri* and *S. sonnei* (around 0.87, Figure 5), it enables discrimination of *E. coli* from the other two species, which is consistent with the data shown in Figure 6. Overall, the five top‐ranked peaks are sufficient for discriminating the different species (*m/z* 1929.7, *m/z* 1783.3, *m/z* 1570.3, *m/z* 1941.9, *m/z* 1950.3) (Table A1).

## 4 DISCUSSION

MALDI‐TOF‐MS is a valuable tool already in use in many clinical microbiology laboratories for rapid species identification directly from bacterial colonies. This project used lipid profiling to investigate whether lipid typing can help to discriminate between the closely related species *E. coli* and *Shigella* spp.

Other methods have been attempted to accurately discriminate *E. coli* from *Shigella*, including protein‐based MALDI‐TOF MS and molecular methods, but due to their close relationship, these assays are not reliable. Currently, identification methods that are routinely used in clinical microbiology laboratories rely on a biochemical characterization using an API20E strip that induces 24 h delay to discriminate *E. coli* from *Shigella* spp. and on subsequent serotyping for *Shigella* (Devanga Ragupathi et al., 2018). Serotyping is based on the O‐antigen expressed on the microbial surface (Allison & Verma, 2000; Gentle et al., 2016; Liu et al., 2021; Sun et al., 2011, 2012). This O‐antigen polysaccharide is the outermost portion of LPS, and its variability among Gram‐negative bacteria allows many pathogens to be classified into serotypes. Serotyping done by agglutination tests is labor‐intensive and errors due to serological cross‐reactivity might occur (Muthuirulandi Sethuvel et al., 2017). Lipopolysaccharide, used for serotyping, is composed of the lipid A, inner core, outer core, and O‐antigen. There is a large similarity between the O‐antigen of *E. coli* and *Shigella* causing such cross‐reactivity. However, in the approach described here, we treat the bacteria with acetic acid 1% v/v for 15 min, which leads to the enrichment of lipids including lipid A, as that treatment cleaves the LPS at the inner core. The remaining products of the hydrolysis are then washed and directly deposited onto the MALDI target plate. This is then overlaid with the matrix which contains apolar solvent (e.g., chloroform), favoring the on‐target extraction and ionization of apolar molecules such as phospholipids and glycolipids. We cannot rule out that the peaks we observed are degradation products of larger molecules allowing the actual discrimination between *Shigella* and *E. coli* despite the high level of similarity between their O‐antigens.

In this context, our results suggested that MALDI‐TOF MS profiling of lipids could provide a straightforward alternative method of species discrimination using the existing routine MALDI mass spectrometer system compared to serotyping which could lead to cross‐reactivity and error in the interpretation of the bacterial strain identification. Indeed, MALDI‐TOF MS is a technique that uses inexpensive reagents and produces robust and reproducible results in a short timeframe. Lipid profiling of bacterial species is an emerging field of research that has already been developed for the rapid identification of antimicrobial resistance traits such as polymyxin resistance using the MALDI‐TOF negative mode (Dortet, Bonin, et al., 2018; Dortet et al., 2019, 2020; Dortet, Tande, et al., 2018; Furniss et al., 2019; Jeannot et al., 2021; Potron et al., 2019). To include this new field of investigation, a specific module has been added to the routine MALDI‐TOF MS of Bruker, the MALDI Biotyper Sirius® system. Accordingly, the implementation of this negative mode paved the way for the development of new methods for bacterial identification.

We should acknowledge that more research is needed before this method of discrimination between *E. coli, S. sonnei*, and *S. flexneri* becomes a gold standard in clinical microbiology laboratories. Indeed, to continue this study, it will be necessary to accumulate data on clinical isolates of *S. dysenteriae* and *S. boydii* that are less prevalent than *S. sonnei* and *S. flexneri*. For a comprehensive analysis, all four serogroups must be represented to validate lipid profiling as a reliable method of bacterial species identification. The bioinformatics analysis pipeline should be repeated with the new data set to determine whether the peaks with the best predictive ability to discriminate between *E. coli, S. sonnei*, and *S. flexneri* also work when *S. boydii* and *S. dysenteriae* are added to the list. In addition, not all *m/z* peaks observed in this study have a confirmed molecule assignment. Future work might also be performed to characterize these newly identified molecules. Of note, another limitation resides in the fact that not all mass spectrometers dedicated to routine microbiology laboratories possess the negative ion mode. However, a module dedicated to lipid profiling has been recently marketed by Bruker on its MALDI Biotyper Sirius® system.

However, this study is another proof‐of‐concept demonstrating that the lipid profiling performed on a routine MALDI‐TOF MS machine with negative ion mode might be a reliable tool for the rapid identification of relevant pathogens. This method can address the needs of clinical microbiology diagnostics that are not met by other assays and help to determine effective treatment options.

## AUTHOR CONTRIBUTIONS

**Jade Pizzato**: Data curation (equal); Investigation (equal); Methodology (equal). **Wenhao Tang**: Data curation (equal); Methodology (equal); Software (equal); Visualization (equal). **Sandrine Bernabeu**: Resources (supporting). **Rémy A. Bonnin**: Resources (supporting). **Emmanuelle Bille**: Resources (supporting). **Eric Farfour**: Resources (supporting). **Thomas Guillard**: Resources (supporting). **Olivier Barraud**: Resources (supporting). **Vincent Cattoir**: Resources (supporting). **Chloe Plouzeau**: Resources (supporting). **Stéphane Corvec**: Resources (supporting). **Vahid Shahrezaei**: Software (supporting). **Laurent Dortet**: Conceptualization (equal); Resources (supporting); Writing – review & editing (supporting). **Gerald Larrouy‐Maumus**: Conceptualization (lead); Funding acquisition (lead); Methodology (supporting); Project administration (supporting); Resources (lead); Writing – original draft (lead); Writing – review & editing (lead).

## CONFLICT OF INTEREST

None declared.

## ETHICS STATEMENT

None required.

## Data Availability

All data are provided in full in the results section of this paper.

## APPENDIX A

**Table A1:** List of the peaks across the data set

Mass (*m/z*)	Score	*Escherichia coli*	*Shigella flexneri*	*Shigella sonnei*
1929.69	131.9508457	−6.038981248	−5.75985843	11.48488106
1783.32	119.0619227	−10.86643176	4.259367801	6.387783086
1570.33	112.8114071	−10.59266041	4.317060838	6.065743451
1941.95	112.6310512	−6.116506281	−4.754975576	10.57899253
1950.28	109.1051441	−5.440223775	−5.289387568	10.44440321
1768.06	107.6628264	−10.20265319	3.154456248	6.822344453
1906.79	106.3185766	−9.96727201	2.381014354	7.349054361
1900.71	105.8585916	−7.252362649	−3.006642121	9.974395202
1847.52	104.1481349	−6.208543489	−4.206108104	10.1324256
1191.16	103.8469808	9.766823143	−2.050572236	−7.477139231
1738.90	94.50000137	−9.430647846	2.375982588	6.83314371
1860.51	94.16492187	−9.564475993	3.073593428	6.281909624
1965.43	92.79566516	−8.114855775	−0.741911486	8.600292379
1796.84	92.07048941	−9.54081868	3.606602853	5.738537977
1823.06	90.8773293	−9.420943619	3.165953545	6.052426461
787.74	89.13091463	9.099844155	−2.082105531	−6.799011212
1891.03	82.56728787	−8.265885652	0.578302393	7.457866682
1887.57	82.00433047	−8.554197352	1.421705108	6.914221587
1041.27	81.96212485	−9.006234984	3.441801825	5.38060058
1920.16	79.37775636	0.155919238	−7.925181001	7.586528703
797.03	79.27845909	8.817398235	−3.074217475	−5.556219971
1703.37	76.90024532	−8.056291753	0.752818945	7.08405261
1348.35	73.93838956	−8.377346812	2.248529199	5.935297528
659.04	71.95044164	6.403872211	1.877681225	−8.04861208
2011.81	70.57446681	−7.277210167	−0.261828809	7.318575734
1749.35	68.05198179	−8.164703393	2.817818437	5.173082111
709.02	66.71906075	8.168170482	−3.852118726	−4.166594128
1164.81	66.24083612	8.026292016	−2.602541196	−5.248935027
1025.44	65.70844827	−8.106016612	3.802032235	4.155172972
1307.40	65.25259846	−6.329376492	−1.43398348	7.543099602
620.68	62.51007603	7.848020253	−4.552013415	−3.172519009
1321.91	62.11313861	−6.534649221	−0.796479402	7.119892485
757.80	61.87438315	6.984999029	−0.111362949	−6.670597847
1292.74	60.14438021	−5.863888875	−1.703428727	7.354394566
770.40	59.82323097	6.972673882	−0.34335082	−6.432131134
1983.75	59.09504227	−5.765064393	−1.758776097	7.31251908
1587.25	57.31923618	−7.557437815	3.968417097	3.460294759
727.48	56.8724406	6.814034765	−0.370728887	−6.251432138
957.23	55.81659636	6.707180998	−0.267692351	−6.248325744
1361.88	55.32288833	−7.427513835	3.161664635	4.121879946
1271.85	54.11528566	3.220337057	−7.350751473	4.051489438
699.09	53.36515186	6.315059854	0.253587678	−6.376709209
2034.93	52.85994191	−7.041881316	1.732132179	5.143345443
1389.50	51.14409368	−6.866312727	5.005807543	1.776649468
1718.46	47.84126365	−6.632907222	1.404752072	5.066055392
836.46	47.23087557	5.431491271	1.145560562	−6.390046656
810.61	46.87506391	6.436019574	−0.982075809	−5.28765067
685.71	46.09456859	5.922449769	0.128175279	−5.873211288
938.87	45.59090603	6.25799246	−0.721408807	−5.369371222
877.29	45.40172959	6.695638032	−2.497333401	−4.060182657
601.92	45.03811116	6.665285397	−3.838095465	−2.721652684
1335.90	44.50511656	−6.376372615	1.283305042	4.935650782
1016.63	43.84154245	−6.579102313	2.450037081	3.993256682
1281.41	42.14957071	−4.524260229	−1.966842437	6.311398259
649.64	40.44688837	6.29733633	−2.191096221	−3.972607573
858.74	37.22540606	6.020500029	−1.971861011	−3.917975847
1056.56	37.19225103	−5.99332018	1.836900602	4.023366708
1688.11	36.06732161	−5.839944303	1.523955277	4.180055293
677.30	35.67840673	5.755312919	−1.309883062	−4.30692813
740.79	35.58782151	4.872902131	0.731314452	−5.443450191
1032.63	35.28684767	−5.94005061	2.760602775	3.069796609
611.45	35.20116134	5.43067702	−0.459379294	−4.822251787
1070.93	34.35533817	−5.750731408	1.717605685	3.904396059
1416.48	34.33625787	−5.046751236	5.008484386	0.008050658
892.863	33.70326354	5.39594765	−0.661208598	−4.591486256
1266.89	30.63769358	−2.880213275	−2.805584165	5.534676359
1075.29	30.08496962	5.476635219	−2.85355838	−2.529267843
1375.92	28.35110789	−5.09764732	3.763501726	1.273003676
985.66	27.8868372	2.116501115	3.264501709	−5.241521705
666.74	26.74663678	5.015463211	−1.257175171	−3.640325056
844.92	25.50277238	4.643752789	−0.444451409	−4.073073262
1430.48	24.86886124	−2.507074473	4.983854044	−2.432798185
819.62	23.24160919	4.378333574	−0.289680593	−3.966581774
1455.85	22.37275097	−1.488983538	4.660398141	−3.105101327
992.34	20.194206	−4.454314505	2.62800179	1.757274157
1123.67	19.5680345	1.588235055	2.888709337	−4.361900294
1472.56	18.92343858	−0.711879596	4.118757222	−3.330485369
1002.52	18.51233603	−3.094698129	4.096515097	−0.996111565
1059.06	16.7718904	−4.045772923	1.35117591	2.607408021
1403.29	15.40490567	−2.647082001	3.804625939	−1.145557917
1079.45	15.18140571	−3.896013599	1.796461816	2.027297422
1443.85	14.59486965	−3.784255251	1.325991789	2.378179486
1095.04	14.37074513	3.53679869	−2.873349307	−0.627225878
908.78	12.46917465	−0.83257007	3.417903098	−2.529060236
896.62	12.43679004	−3.12681473	0.039570725	2.996111526
924.37	11.25711229	1.201184542	2.193802994	−3.307765181
976.95	10.51236242	0.618289425	−3.097355813	2.424060825
1108.39	7.418772384	−1.968568646	2.589066509	−0.617264936
1136.41	6.434015026	2.311432716	−0.171117322	−2.076300819

*Note*: The 5 top‐ranked peaks are highlighted in grey.
